# HLA DRB1*01 and *04 Predisposition to Rheumatoid Arthritis and Polymorphisms of the *SLCO1B1*, *MTHFR* and *PNPLA3* Genes Are Not Associated with Fatty Liver and Hepatotoxicity

**DOI:** 10.3390/jcm15041568

**Published:** 2026-02-16

**Authors:** Tatjana Zekić, Nataša Katalinić, Nada Starčević Čizmarević, Aleksandar Čubranić

**Affiliations:** 1 Department of Rheumatology and Clinical Immunology, Clinical Hospital Center Rijeka, 51000 Rijeka, Croatia; 2 Clinical Institute for Transfusion Medicine, Clinical Hospital Center Rijeka, Faculty of Medicine, University of Rijeka, 51000 Rijeka, Croatia; nkatalinic1@gmail.com (N.K.); acubrani@yahoo.com (A.Č.); 3 Institute for Medical Biology and Genetics, Faculty of Medicine, University of Rijeka, 51000 Rijeka, Croatia; nadasc@medri.uniri.hr; 4 Department of Gastroenterology and Hepatology, Clinical Hospital Center Rijeka, Faculty of Medicine, University of Rijeka, 51000 Rijeka, Croatia

**Keywords:** *HLA-DR* antigens, methylenetetrahydrofolate reductase, methotrexate, non-alcoholic fatty liver disease, patatin 3-like phospholipase domain-containing protein, rheumatoid arthritis, soluble transporter of organic anions 1B1

## Abstract

**Background**: Nonalcoholic fatty liver disease (NAFLD) is common in rheumatoid arthritis (RA), and methotrexate (MTX) use raises concern about hepatotoxicity. We evaluated whether *HLA-DRB1*, *PNPLA3*, *SLCO1B1*, and *MTHFR* variants are associated with NAFLD, liver fibrosis, or MTX toxicity/pharmacokinetics in RA, after accounting for clinical covariates. **Methods**: In a cross-sectional cohort of 159 patients with RA, NAFLD, and fibrosis were assessed by FibroScan (CAP ≥ 275 dB/m; LSM > 8 kPa). We compared baseline characteristics by NAFLD status and fitted multivariable models for NAFLD, fibrosis, ALT elevation, and MTX toxicity; MTX pharmacokinetics were analyzed in 111 MTX-treated patients. Multiple testing was controlled using the Benjamini–Hochberg method. **Results**: The prevalence of NAFLD was 36%, and that of fibrosis was 11%. NAFLD patients had higher CAP and LSM, and markedly greater adiposity indices (body weight, BMI, waist and hip circumference, WC). BMI and WC were independently associated with NAFLD (BMI OR 1.27 per kg/m^2^, 95% CI 1.16–1.40; WC OR 1.06 per cm, 95% CI 1.01–1.12). No *HLA-DRB1*, *PNPLA3*, *SLCO1B1*, or *MTHFR* variant showed an association that survived multiple-comparison correction. Among MTX users, 21/111 (19%) experienced toxicity. *SLCO1B1* and *MTHFR* variants did not influence MTX pharmacokinetics; age was associated with lower dose-normalized MTX exposure, and cumulative dose was positively associated with exposure. **Conclusions**: In RA, adiposity—not the tested candidate pharmacogenes—drives NAFLD risk, and *SLCO1B1*/*MTHFR* variants do not support MTX dose adjustment. The findings emphasize routine clinical risk factors over single-gene testing for NAFLD and MTX hepatotoxicity in this setting.

## 1. Introduction

**Figure 1:**
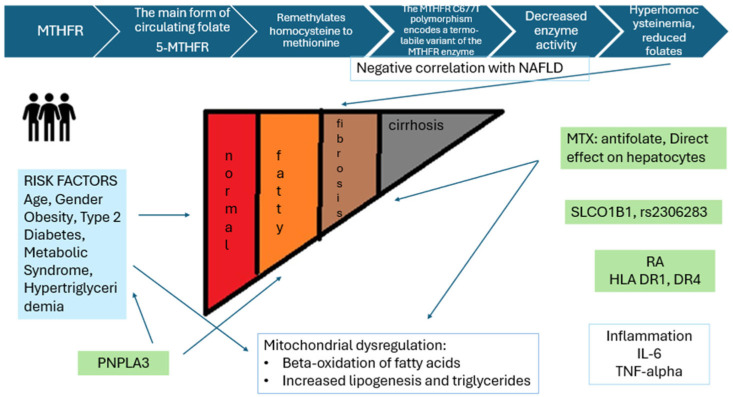
Multifactorial origin of NAFLD in RA patients.

Rheumatoid arthritis (RA) is a chronic, autoimmune disease that, if untreated, leads to progressive joint damage and disability. Women are more likely to be affected than men, with a female-to-male ratio of approximately 2–3:1 [1]. The prevalence of RA globally is ~0.5–1% [2]. RA is strongly associated with major histocompatibility complex class II (MHC II) human leukocyte antigen (*HLA*)-DR alleles, as well as non-*HLA* loci [3]. Among these, *HLA–DRB1* shared epitope (SE) alleles are the strongest genetic risk factors and are linked to anti-citrullinated protein antibody (ACPA)-positive disease [4]. Methotrexate (MTX) is the first-line standard therapy for RA. Long-term MTX use has been associated with nonalcoholic fatty liver disease (NAFLD), nonalcoholic steatohepatitis (NASH), and hepatic fibrosis [5]. Methylenetetrahydrofolate reductase (*MTHFR*) and solute carrier organic anion transporter family member 1B1 (*SLCO1B1*) have been implicated in MTX pharmacokinetics. The *MTHFR* C677T (rs1801133) variant has been investigated in relation to MTX efficacy and toxicity in RA and, separately, to NAFLD susceptibility [6]. However, evidence linking C677T to MTX toxicity remains inconsistent. Hepatic uptake of MTX is mediated in part by OATP1B1 (encoded by *SLCO1B1*) [7]. Reports on associations between *SLCO1B1* variants and MTX toxicity or clearance are mixed across studies [8]. Interindividual variability in MTX pharmacokinetics complicates toxicity prediction, and relationships among route of administration, dose, and bioavailability are nonlinear, especially at higher doses [9]. Among NAFLD risk variants, patatin-like phospholipase domain-containing 3 (*PNPLA3*) rs738409 C>G (p.I148M) is a major determinant of hepatic steatosis and fibrosis through lipid accumulation and lipotoxicity [10]. To our knowledge, *PNPLA3* has not been systematically evaluated in RA cohorts with FibroScan-defined NAFLD. Here, we present a study evaluating candidate pharmacogenes and NAFLD in an RA cohort characterized by FibroScan. We test the hypotheses that patient anthropometrics and cumulative MTX exposure associate with NAFLD, and that polymorphisms in *HLA-DRB1*, *PNPLA3*, *SLCO1B1*, and *MTHFR* are risk factors for NAFLD and MTX toxicity, as outlined in Figure 1.

## 2. Materials and Methods

### 2.1. Study Design and Setting

This cross-sectional study was conducted in the Department of Rheumatology at a tertiary hospital center. We compared patients with rheumatoid arthritis (RA) who had nonalcoholic fatty liver disease (NAFLD) with those who did not, and examined correlations between anthropometric and laboratory measures and the controlled attenuation parameter (CAP) to assess the influence of patient characteristics on NAFLD. We also evaluated the relationships of disease activity and HLA-DRB1*01 and *04 with CAP, the association of cumulative methotrexate (MTX) dose with CAP, and whether selected genetic variants were associated with NAFLD, gastrointestinal (GI) toxicity, or elevated liver function tests (alanine aminotransferase, ALT).

### 2.2. Participants and Outcomes

All participants fulfilled the 2010 ACR/EULAR classification criteria for RA and underwent FibroScan assessments. NAFLD was defined as CAP ≥ 275 dB/m, and liver fibrosis as a liver stiffness measurement (LSM) > 8 kPa. Among MTX-treated patients, toxicity events and ALT elevation (>36 U/L) were recorded. Pharmacogenetic analyses were performed in all patients; MTX pharmacokinetics were evaluated in the subset of MTX users. Patients were recruited in 2023 and 2024 after ethical approval. Of the 159 patients, 20 had fibroscan data before 2023 and were subsequently included only in the genetic analysis. The other patients underwent laboratory tests, then fibroscan, and finally pharmacogenetic analysis.

### 2.3. Eligibility Criteria

Adults (≥18 years) were eligible if they met the ACR/EULAR classification criteria for RA and had been on unchanged methotrexate therapy for at least 6 months. To reduce confounding from clinical dose adjustment and altered methotrexate clearance, participants were excluded if they had clinically relevant renal impairment at the time of assessment (based on serum creatinine and estimated glomerular filtration rate) or severe RA-associated lung disease (including clinically significant interstitial lung disease). Additional exclusion criteria were alcohol intake >20 g/day, viral hepatitis, autoimmune liver disease, current or prior malignancy, thyroid disease, pregnancy, positive antinuclear antibodies (ANA), regular use of non-steroidal anti-inflammatory drugs, or missing/incomplete data.

### 2.4. Genotyping

Genetic analyses targeted *MTHFR* (rs1801133), *SLCO1B1* (rs4149081), and *PNPLA3* (rs738409[G], encoding p.I148M), as well as HLA-DRB1*01 and *04.

Molecular *HLA* typing used polymerase chain reaction–sequence specific primer (PCR-SSP) on device SimpliAmpTM Thermal Cycler (Applied Biosystems by Thermo Fisher Scientific Inc., Waltham, MA, USA) and PCR-SSO (engl. Sequence-Specific Oligonucleotides, SSO) on device Luminex 200TM (Luminex Corp., Austin, TX, USA). We used commercial kits, Olerup SSP^®^ DR low resolution (CareDx AB, Stockholm, Sweden), the LIFECODES *HLA DRB1* SSO Typing Kit (Immucor Inc., Norcross, GA, USA). *HLA-DRB1* alleles were typed at low resolution by PCR-SSP. *SLCO1B1* rs4149081 was determined by PCR, and *MTHFR* rs1801133 and *PNPLA3* rs738409 by PCR–restriction fragment length polymorphism (PCR-RFLP) (restriction enzymes New England Biolabs, Ipswich, MA, USA).

### 2.5. Ethics

The study adhered to the principles of the 1975 Declaration of Helsinki. It was approved by the Ethics Committee of the Clinical Hospital Center Rijeka (approval no.: 2170-29-02/1-23-2; date 23 November 2023) and the Faculty of Medicine of the University of Rijeka (approval no.: 2170-1-42-04-36/1-24-5; date 30 April 2024). All participants provided written informed consent.

Microsoft 365 Copilot was used to generate a graphical abstract according to our key points; date 7 January 2026.

### 2.6. Statistics

Baseline characteristics were summarized for the overall cohort and compared between patients with and without NAFLD (CAP ≥ 275 dB/m). Continuous variables are reported as mean ± standard deviation (SD) and compared using the t-test or Wilcoxon rank-sum test, as appropriate. Categorical variables are presented as counts and percentages and compared using the chi-square test or Fisher’s exact test. Two-sided *p*-values < 0.05 were considered statistically significant. Analyses were performed in R (version 4.3.3; R Foundation for Statistical Computing, Vienna, Austria).

Associations of genetic polymorphisms (*HLA*-*DRB1* alleles, *PNPLA3*, *SLCO1B1*, and *MTHFR*) with clinical outcomes were examined in multivariable logistic regression models in the full cohort (No= 159). Outcomes included NAFLD (CAP ≥ 275 dB/m), liver fibrosis (LSM > 8 kPa), ALT elevation (>36 U/L), and MTX toxicity; effects on MTX pharmacokinetics were assessed in MTX-treated patients (No = 111). Given the number of markers and endpoints, *p* values were adjusted using the Benjamini–Hochberg procedure, and interpretation prioritized adjusted *p* values. For MTX toxicity, Firth’s penalized logistic regression was used due to the limited number of events (21/111), providing more stable estimates than standard logistic regression in sparse data settings.

To assess whether *PNPLA3* polymorphisms contribute to NAFLD development in non-obese individuals, we performed stratified and interaction analyses. First, we restricted analysis to patients with normal BMI (<25 kg/m^2^, No = 63) and used Fisher’s exact test to compare *PNPLA3* genotype distribution between those with and without NAFLD. Fisher’s exact test was selected due to small expected cell frequencies. Second, we tested whether obesity status (BMI ≥ 30 kg/m^2^) modifies *PNPLA3*’s effect using multivariable logistic regression with an interaction term. We fitted two nested models: (1) a main effects model including *PNPLA3* genotype, obesity status, age, and gender, and (2) an interaction model additionally including *PNPLA3* × obesity interaction. The interaction’s significance was assessed using a likelihood ratio test comparing the models. The interaction term had 2 degrees of freedom, corresponding to GC vs. GG and CC vs. GG contrasts tested for differential effects across obesity strata.

## 3. Results

### 3.1. Cohort and Baseline Characteristics

Among 159 patients with RA, 58 (36%) met criteria for NAFLD (CAP ≥ 275 dB/m) and 18 (11%) had fibrosis (LSM > 8 kPa). As expected, CAP and LSM were higher in the NAFLD group (both *p* ≤ 0.017). Anthropometric indices diverged markedly; patients with NAFLD had higher body weight, BMI, waist circumference, and hip circumference (all *p* < 0.001), and obesity (BMI ≥ 30 kg/m^2^) was more common (50.0% vs. 9.9%, *p* < 0.001). Lipids and enzymes reflected greater metabolic risk in NAFLD (higher triglycerides, *p* = 0.012; higher ALT, *p* < 0.001). No between-group differences were observed for age, sex, disease duration, RF or anti-CCP, AST, CRP, glucose, DAS28-CRP, or MTX/prednisone exposure (all *p* > 0.05). Summary statistics are provided in Table 1.

### 3.2. Genetics and NAFLD

#### 3.2.1. *HLA*-*DRB1* Alleles and NAFLD

We modeled NAFLD (CAP ≥ 275 dB/m) as the dependent variable in multivariable logistic regressions for HLA-DRB1*01, HLA-DRB1*04, HLA-DRB1*03, and HLA-DRB1*07 (covariates: age, sex, BMI).

Across models, BMI was a robust risk factor (Table 2), whereas age and sex were not significant. After Benjamini–Hochberg correction, none of the *HLA*-*DRB1* alleles showed an independent association with NAFLD; *HLA*-DRB1*04 displayed a nonsignificant trend toward lower odds of NAFLD (illustrative OR ≈ 0.70; 95% CI spanning 1). These results indicate that, within this sample, anthropometrics dominate over the tested *HLA* risk alleles for NAFLD classification.

#### 3.2.2. *PNPLA3*, *SLCO1B1*, and *MTHFR*: Associations with NAFLD and Fibosis

We fitted minimally and fully adjusted models to evaluate associations between candidate genotypes and hepatic outcomes (Table 3).

For *PNPLA3*, in the minimally adjusted logistic model (age, sex, BMI), neither GC nor CC differed from the GG reference for NAFLD risk. BMI remained a strong independent predictor (OR = 1.27 per kg/m^2^; 95% CI, 1.16–1.40; estimate = 0.24; SE = 0.05; z = 4.90; *p* < 0.001). In the fully adjusted model (also including waist circumference, triglycerides, total cholesterol, glucose, cumulative MTX dose, and prednisone), *PNPLA3* genotypes again showed no association; waist circumference was independently related to NAFLD (OR = 1.06 per cm; 95% CI, 1.01–1.12; estimate = 0.06; SE = 0.03; z = 2.31; *p* = 0.021), while other covariates were not significant.

For *SLCO1B1* and *MTHFR*, linear models with CAP (dB/m) as the continuous outcome showed no genotype effects that survived multiple-testing correction. *MTHFR* C677T (CT) carried a negative estimate for CAP in the minimal model (β = −18.7 dB/m; 95% CI, −35.6 to −1.86; *p* = 0.030; FDR-adjusted *p* = 0.119) and in the fully adjusted model (β ≈ −19.5 dB/m; *p* ≈ 0.036; adjusted *p* = 0.351), but these did not remain significant after false-discovery-rate control. Logistic models for fibrosis (LSM > 8 kPa) likewise showed no significant genotype associations; confidence intervals were wide, consistent with sparse events.

#### 3.2.3. *SLCO1B1* Genotype Does Not Influence Methotrexate Pharmacokinetics

Among current MTX users (No = 111), dose-normalized cumulative MTX exposure did not differ by *SLCO1B1* genotype (AA, AG, GG; Kruskal–Wallis χ^2^ = 0.18, df = 2, *p* = 0.913). Post hoc Wilcoxon tests with Benjamini–Hochberg adjustment showed no differences between any genotype pairs (all adjusted *p* = 0.91).

The results were similar within NAFLD strata. In MTX users with NAFLD (No = 45), the *SLCO1B1* genotype was not associated with dose-normalized exposure (χ^2^ = 1.27, df = 2, *p* = 0.530; all adjusted *p* = 0.54). In those without NAFLD (No = 66), the Kruskal–Wallis test was again non-significant (χ^2^ = 0.98, df = 2, *p* = 0.613; adjusted *p* = 0.66–0.89).

A multivariable linear model of log-transformed dose-normalized exposure, including *SLCO1B1* genotype, NAFLD status, genotype-by-NAFLD interaction, age, sex, and BMI, confirmed the absence of association (genotype overall F = 0.70, *p* = 0.500; NAFLD F = 1.17, *p* = 0.282; interaction F = 0.19, *p* = 0.829). Age showed a borderline association (*p* = 0.074); other covariates were not significant. Together, these analyses indicate that *SLCO1B1* polymorphisms do not affect MTX pharmacokinetics in this cohort.

#### 3.2.4. *MTHFR* C677T and NAFLD

In MTX users (No = 111), dose-normalized MTX exposure did not differ across *MTHFR* C677T genotypes (CC, CT, TT) in the Kruskal–Wallis tests, stratified analyses, or gamma regression (all non-significant after adjustment), suggesting limited clinical utility of *MTHFR* C677T for MTX dose prediction in RA, irrespective of NAFLD status. Age was associated with lower dose-normalized exposure (estimate = −0.0085, SE = 0.0038, t = −2.24, *p* = 0.028), and cumulative MTX dose was positively associated with exposure (estimate = 3.34 × 10^−5^, SE = 8.34 × 10^−6^, t = 4.00, *p* = 0.0001), as expected.

Notably, the observed negative estimates for *MTHFR* CT and TT versus CC in CAP-based models (reported above) suggest a potential protective trend for hepatic steatosis; however, these did not survive multiple-testing correction and require validation in larger cohorts.

#### 3.2.5. Effect on ALT

Fisher’s exact tests showed no association between genotype and ALT elevation >36 U/L for *PNPLA3* (*p* = 0.176, adjusted *p* = 0.301), *SLCO1B1* (*p* = 0.201, adjusted *p* = 0.301), or *MTHFR* C677T (*p* = 0.834, adjusted *p* = 0.834).

**Figure 2:**
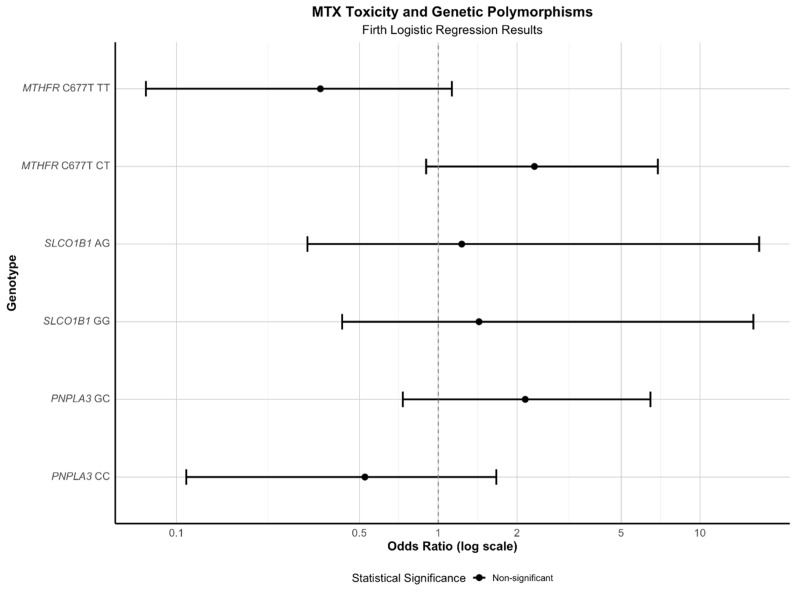
Forest plot (log scale) of odds ratios with 95% CIs for the association between PNPLA3, SLCO1B1, and MTHFR C677T genotypes and risk of any MTX-related toxicity among MTX users (No = 111), estimated with Firth’s penalized logistic regression. All CIs cross 1.0; no genotype term remained significant after adjustment for multiple comparisons. Abbreviations: SLCO1B1—solute carrier organic anion transporter family member 1B1; MTHFR—Methylenetetrahydrofolate reductase; PNPLA3—patatin-like phospholipase do-main-containing 3; MTX—methotrexate; CI—confidential interval.

After multiple-testing correction, none of the three polymorphisms was associated with ALT elevation >36 U/L (all adjusted *p* > 0.30). Power may have been limited by the small number with elevated ALT (No = 20), but effect estimates were weak across all genotypes. These findings suggest that the tested variants are not major determinants of ALT elevation in this rheumatology cohort (Figure 2).

#### 3.2.6. *PNPLA3* Genotype and Lean NAFLD

Among 63 patients with normal BMI (<25 kg/m^2^), 8 (12.7%) had NAFLD. Fisher’s exact test revealed no significant association between *PNPLA3* genotype and NAFLD in lean patients (*p* = 0.430). To formally test whether obesity modifies *PNPLA3*’s effect, we fitted a logistic regression model including a *PNPLA3* × obesity interaction term, adjusting for age and gender. Sequential analysis of deviance showed that the interaction term was not significant (χ^2^ = 1.53, df = 2, *p* = 0.466), indicating that *PNPLA3*’s association with NAFLD does not differ between obese and non-obese patients. These findings should be interpreted with caution, given their limited statistical power (only eight lean NAFLD cases).

## 4. Discussion

NAFLD in our cohort was strongly associated with increased adiposity, central obesity, higher triglyceride levels, and elevated ALT, but not with demographic or RA-specific characteristics. No statistically significant associations were observed between *PNPLA3*, *SLCO1B1*, or *MTHFR* genotypes and NAFLD risk, irrespective of covariate adjustment. The *HLA* risk genes for RA (*HLA*-DRB1*01 and HLA-DRB1*04) did not affect fatty liver in our data; the latter may warrant further study. The relationship between *HLA* and NAFLD is an emerging area in immunology and hepatology. *HLA* molecules are central to antigen presentation and immune regulation, and variation in *HLA* genes may influence susceptibility to several liver diseases, including NAFLD. In a study of 140 participants (85 biopsy-confirmed NAFLD, 55 controls), *HLA*-*DRB1∗03* was linked to a higher risk of NASH, and *HLA*-*B65* was independently associated with NAFLD; *HLA*-*DRB1∗07* also conferred increased NAFLD risk [11]. In our study, *HLA*-*DRB1*∗03 and ∗07 were not significant. Few studies have analyzed *HLA*-*DRB1* in NAFLD. One investigation reported that HLA-B*54:01 increased NAFLD risk and might influence disease through microbiome regulation; *HLA*-*DRB1* was also examined, but sequencing failed in that analysis [12].

### 4.1. PNPLA3

Across both of our models, the *PNPLA3* genotype was not associated with NAFLD, and no variant remained significant after multiple-testing correction (all adjusted *p* > 0.24). Point estimates for GC and CC genotypes were near unity (OR 1.36 and 1.04 in the minimally adjusted model), with wide confidence intervals crossing 1.0, indicating no association and considerable uncertainty, likely reflecting limited power. In both models, BMI (minimally adjusted) and waist circumference (fully adjusted) were consistent, strong predictors of NAFLD, underscoring the central role of adiposity. *PNPLA3* is highly expressed in the liver and participates in triglyceride hydrolysis, regulating lipid storage and mobilization. Variants can alter enzyme activity, affecting hepatic fat accumulation and NAFLD onset. The I148M (rs738409) variant is the most extensively studied and is linked to increased risks of NAFLD and fibrosis; carriers accrue more hepatic fat and have higher rates of liver-related complications [13]. A single variant may not capture overall genetic risk. For example, carriers of one *PNPLA3* rs738409-G allele and two TM6SF2 rs58542926-T alleles have a higher NAFLD risk [14]. Genotyping may be informative in select patients (e.g., young, lean individuals), as carriers of *PNPLA3* p.I148M and TM6SF2 p.E167K can have persistently elevated liver fat and an increased risk of NASH [15]. Population differences also contribute: the frequency of the G minor allele is ~49% in Hispanics, 23% in Europeans, and 17% in African Americans [13,16]. In our cohort, the genotype frequencies were GG: 6%; GC: 35%; and CC: 59%.

### 4.2. SLCO1B1

Polymorphisms in *SLCO1B1* (rs4149081) were not associated with fatty liver, MTX pharmacokinetics, or elevated ALT. The role of *SLCO1B1* in NAFLD is little studied, although the rationale is clear: patients with NAFLD often have altered hepatic function, and *SLCO1B1* encodes OATP1B1, a transporter involved in hepatic uptake of bile acids, which are integral to fat digestion and metabolism. Disruption of bile acid transport may promote hepatic fat accumulation and inflammation—key NAFLD features. Certain *SLCO1B1* variants (388A>G, rs2306283; 521T>C, rs4149056) have been linked to differences in triglycerides, HDL-C, and LDL-C—NAFLD risk factors [17]. In 100 patients with JIA, *SLCO1B1* rs4149056 (CT/CC) was associated with higher odds of MTX gastrointestinal side effects, and rs4149056 TT with hepatotoxicity; rs2306283 showed no association [18]. In contrast, a study of 100 RA patients on MTX reported associations between rs2306283 and GI disturbances and anemia [19]. Regarding NASH, findings are mixed: some studies show no change in hepatic OATP expression, others report reduced OATP1B1/1B3/2B1, and another found OATP1B1 overexpression with unaffected OATP2B1 [20]. Overall, evidence is still emerging, and conclusions remain tentative. To date, studies of *SLCO1B1* rs2306283 AA with low-dose MTX are scarce [18,19]. Variants in *SLCO1B1* may also modulate the risk of drug-induced liver injury (DILI) by altering hepatic uptake of potentially hepatotoxic drugs, particularly in patients with NAFLD [21]. *SLCO1B1* (rs4149081) has previously been reported to be associated, in some form, with MTX pharmacokinetics, but not with fatty liver disease or elevated ALT levels. It has also not been studied extensively in relation to hyperlipoproteinemia. A study including 247 patients demonstrated that the intronic SNP rs4149081 in *SLCO1B1* was associated with the LDL-C response to statin therapy in a Chinese population [22]. Due to the high prevalence of MTX intolerance in children, numerous *SLCO1B1* polymorphisms have been investigated in juvenile idiopathic arthritis. The variant most frequently associated with gastrointestinal intolerance is rs4149056 [23]. Understanding *SLCO1B1*’s role in drug handling may support individualized pharmacotherapy in NAFLD. Genetic testing for *SLCO1B1* variants may guide treatment decisions, particularly regarding medications that are substrates of this transporter. The c.388A>G (p.N130D) allele is common in African and Asian populations (~75%) and less common in Europeans (~40%) [24].

### 4.3. MTHFR Polymorphisms and Fatty Liver

*MTHFR* is relevant to MTX pharmacokinetics because MTX and folate share pathways, and *MTHFR* variants can alter folate metabolism, potentially affecting MTX efficacy and toxicity. Homocysteine, an intermediate in hepatic methionine metabolism, may contribute to liver disease through oxidative and ER stress and pro-inflammatory signaling (Figure 1) [6]. In the first meta-analysis of *MTHFR* and NAFLD (785 cases, 1188 controls), higher NAFLD risk (C677T) was observed in the homozygote model (TT vs. CC: OR 1.49, 95% CI 1.03–2.15, *p* = 0.04) and the recessive model (TT vs. CC + CT: OR 1.42, 95% CI 1.07–1.88, *p* = 0.02); susceptibility was also linked to the CC genotype of *MTHFR* A1298C [25]. Findings across studies vary, and many analyses are underpowered. A key clinical question is whether cumulative MTX dose worsens NAFLD and fibrosis in susceptible individuals. Definitive answers will require large trials including patients with and without NAFLD, standardized folate supplementation, and long-term follow-up, with careful control for competing risk factors that influence NAFLD trajectories (e.g., weight change, diabetes, dyslipidemia, and adherence) [26].

### 4.4. Hepatoxicity

In a Spanish study (No = 53), the *MTHFR* C677T T allele (*p* = 0.006) and ABCB1 C3435T C allele (*p* = 0.046) were associated with toxicity after 12 months of MTX therapy [27]. In early RA (No = 54), the C677T T allele was associated with MTX side effects in the first 6–12 months (*p* = 0.019; OR 3.63, 95% CI 1.12–12); 16% experienced side effects, including 44% gastrointestinal and 22% hepatic events [28]. Conversely, a GWAS interrogating *MTHFR* C677T (rs1801133) and the *HLA* region did not identify significant signals [29]. Cumulative MTX dose and treatment duration are major determinants of MTX-induced hepatotoxicity. Additional contributors include concomitant drugs or chemicals, age, viral hepatitis (B/C), and family history. Genetic markers may help identify patients at risk. Historically, *MTHFR* C677T was the primary candidate marker for low-dose MTX hepatotoxicity, but additional polymorphisms may be relevant. Future research should include adequately powered GWAS to uncover novel markers [30]. A recent meta-analysis of *PNPLA3* and hepatotoxicity (five studies; No = 3480) found no significant association between I148M carriage and clinically relevant hepatocellular DILI; the number of studies remains small [31].

Factors potentially affecting pharmacologic efficacy include patient-related (age, gender, ethnicity, comorbidities), disease-related (duration, activity, disability, biomarkers), treatment-related (adherence, dosage, prior medications), and genetic factors [32]. Drug toxicity is influenced by exposure, environmental conditions, and host factors [33].

In our analyses, which used standard and Firth-penalized logistic regression, no genotype (*PNPLA3*, *SLCO1B1*, or *MTHFR* C677T) was significantly associated with MTX-related toxicity (including elevated ALT), whether defined broadly or by specific phenotypes. Although some point estimates suggested increased or decreased risk (with *MTHFR* C677T TT notably showing a possible protective trend), wide confidence intervals and a lack of significance after multiple-testing correction warrant caution when drawing conclusions (Figure 2).

Genetic studies in the area of adverse events frequently have small sample sizes (<300). To evaluate independent associations of demographic, clinical, and genetic factors with response or adverse events, multivariable analyses with careful confounder control are needed; however, some studies rely on univariate approaches. In addition, the relationship among route of administration, dose, and MTX bioavailability is nonlinear, particularly at higher doses [9].

### 4.5. Clinical Application of Pharmacogenomic Testing

Several online resources provide clinical guidance based on pharmacogenomic (PGx) results. PGx aims to tailor therapy to the patient’s genetic profile. Test content, the number of drugs covered, and access vary across countries [34]. PharmGKB (Pharmacogenomics Knowledge Base) is an NIH-funded repository that curates clinically relevant gene–drug interactions and genotype–phenotype relationships [35]. A case report used a commercial PGx panel (Stratipharm^®^, Humatrix AG, Pfungstadt, Germany) to contextualize adverse drug reactions after MTX. The panel interrogates multiple MTX-related genes and provides interpretive guidance; it also evaluates the pharmacogenomics of co-medications. Recommendations are based on the published literature and curated databases. In that case, the side effects of MTX were plausibly linked to the patient’s PGx profile; however, the authors were uncertain whether pre-emptive panel testing in primary care meaningfully improves low-dose MTX management [36].

Clinicians often struggle with when and for whom to order testing. Despite pharmacogenomic information appearing on many drug labels, uptake remains limited, owing to gaps in provider knowledge, test costs, time constraints, lack of clear clinical pathways, limited test availability, and ethical considerations [37]. Nonetheless, multigene PGx tests have reduced costs, and groups such as the Clinical Pharmacogenetics Implementation Consortium have issued implementation guidelines [38].

### 4.6. Strengths and Limitations

Strengths: Our gene analyses incorporated covariates—BMI and waist circumference—that showed robust associations with NAFLD. Several results that appeared *significant* without covariate control became non-significant after adjustment, highlighting the importance of confounding control. Data on *SLCO1B1* (rs4149081) in NAFLD are minimal, and with more studies on rs2306283 and rs4149056 [18,19,23]; our work adds evidence relevant to pharmacogenomic panels.

Limitations: The cohort was predominantly female, and the number of adverse events was modest, limiting power for toxicity analyses. MTX metabolism involves many genes beyond those examined here, and the interpretation of genetic effects requires covariate integration. Given NAFLD’s multifactorial etiology and the complexity of polygenic panels, broad generalization is challenging. An individualized approach to genetic testing is likely most appropriate. Apart from the widely studied *MTHFR*, this study provides rare data on *HLA*-*DRB1*, *SLCO1B1*, and *PNPLA3* in a specific clinical setting.

## 5. Conclusions

In this FibroScan-characterized RA cohort, NAFLD was driven primarily by metabolic factors—higher BMI and waist circumference, elevated triglycerides, and higher ALT—rather than by RA-specific characteristics or the candidate pharmacogenes examined. Across complementary models and after correction for multiple testing, we found no evidence that *PNPLA3*, *SLCO1B1*, or *MTHFR* C677T genotypes were associated with NAFLD or with MTX-related toxicity. Likewise, *SLCO1B1* variants did not influence dose-normalized MTX exposure, and *HLA* risk alleles for RA (*HLA*-*DRB1/DR4*) were not associated with fatty liver in this sample. These findings argue against using routine clinical genotyping of these variants to guide MTX dosing or to predict hepatotoxicity in unselected RA populations. Instead, management should prioritize modifiable metabolic risk—weight reduction, triglyceride control, and monitoring of liver enzymes—alongside standard MTX safety practices. Our study is limited by a modest sample size and the resulting uncertainty around small genetic effects. Larger, prospective, and ethnically diverse studies that integrate rigorous phenotyping (including transient elastography metrics), cumulative MTX exposure and route, folate supplementation, and broader genomic approaches (e.g., GWAS and multigene panels) are needed to clarify gene–environment interactions and to identify patients who may benefit from targeted pharmacogenomic testing.

## Figures and Tables

**Table 1:** Baseline demographic, clinical, and laboratory characteristics of the study cohort.

Variable	Overall (N = 159)	Non-NAFLD (N = 101)	NAFLD (N = 58)	*p*-Value	Statistical Test
Age (years)	59.13 ± 9.24	57.99 ± 9.66	61.11 ± 8.17	0.065	Wilcoxon
Gender (Female)	M: 23 (14.5%); F: 136 (85.5%)	M: 15 (14.9%); F: 86 (85.1%)	M: 8 (13.8%); F: 50 (86.2%)	1.000	Chi-square
Disease duration (years)	8.04 ± 6.47	8.27 ± 6.52	7.65 ± 6.41	0.522	Wilcoxon
Disease duration < 2 years	0: 141 (88.7%); 1: 18 (11.3%)	0: 88 (87.1%); 1: 13 (12.9%)	0: 53 (91.4%); 1: 5 (8.6%)	0.579	Chi-square
RF-positive	0: 51 (32.9%); 1: 104 (67.1%)	0: 32 (32.3%); 1: 67 (67.7%)	0: 19 (33.9%); 1: 37 (66.1%)	0.979	Chi-square
Anti-CCP-positive	0: 35 (24%); 1: 111 (76%)	0: 20 (21.5%); 1: 73 (78.5%)	0: 15 (28.3%); 1: 38 (71.7%)	0.469	Chi-square
Fibrosis (LSM ≥8 kPa)	0: 141 (88.7%); 1: 18 (11.3%)	0: 92 (91.1%); 1: 9 (8.9%)	0: 49 (84.5%); 1: 9 (15.5%)	0.315	Chi-square
CAP (dB/m)	260.55 ± 53.71	229.54 ± 36.14	314.55 ± 32.15	<0.001	Wilcoxon
LSM (kPa)	5.46 ± 2.36	5.12 ± 2.08	6.04 ± 2.69	0.017	Wilcoxon
Body weight (kg)	75.28 ± 13.11	71.07 ± 10.76	82.60 ± 13.69	<0.001	Wilcoxon
BMI (kg/m^2^)	27.29 ± 6.80	25.28 ± 3.63	30.79 ± 9.25	<0.001	Wilcoxon
Normal BMI (<25)	0: 96 (60.4%); 1: 63 (39.6%)	0: 46 (45.5%); 1: 55 (54.5%)	0: 50 (86.2%); 1: 8 (13.8%)	<0.001	Chi-square
Overweight BMI (25–30)	0: 102 (64.2%); 1: 57 (35.8%)	0: 65 (64.4%); 1: 36 (35.6%)	0: 37 (63.8%); 1: 21 (36.2%)	1.000	Chi-square
Obese BMI (≥30)	0: 120 (75.5%); 1: 39 (24.5%)	0: 91 (90.1%); 1: 10 (9.9%)	0: 29 (50%); 1: 29 (50%)	<0.001	Chi-square
Waist circumference (cm)	94.33 ± 14.10	89.19 ± 11.59	103.28 ± 13.68	<0.001	Wilcoxon
Hip circumference (cm)	104.90 ± 10.79	101.35 ± 8.27	111.08 ± 11.89	<0.001	Wilcoxon
Triglycerides (mmol/L)	1.66 ± 0.99	1.51 ± 0.84	1.91 ± 1.16	0.012	Wilcoxon
Total cholesterol (mmol/L)	5.77 ± 1.39	5.86 ± 1.36	5.60 ± 1.44	0.266	t-test
ALT (U/L)	25.63 ± 13.07	22.88 ± 11.31	30.36 ± 14.58	<0.001	Wilcoxon
AST (U/L)	23.14 ± 9.07	22.37 ± 8.30	24.45 ± 10.19	0.278	Wilcoxon
CRP (mg/dL)	4.65 ± 5.45	4.40 ± 5.68	5.08 ± 5.06	0.103	Wilcoxon
Glucose (mmol/L)	5.32 ± 1.09	5.15 ± 0.87	5.61 ± 1.35	0.070	Wilcoxon
DAS28-CRP	2.66 ± 1.18	2.60 ± 1.19	2.77 ± 1.15	0.317	Wilcoxon
Currently on MTX	0: 47 (29.6%); 1: 112 (70.4%)	0: 34 (33.7%); 1: 67 (66.3%)	0: 13 (22.4%); 1: 45 (77.6%)	0.188	Chi-square
MTX dose (mg/week)	9.46 ± 7.05	8.88 ± 7.16	10.45 ± 6.81	0.233	Wilcoxon
MTX duration (months)	74.85 ± 287.68	88.51 ± 361.08	52.25 ± 64.66	0.686	Wilcoxon
Prednisone use	0: 68 (42.8%); 1: 91 (57.2%)	0: 44 (43.6%); 1: 57 (56.4%)	0: 24 (41.4%); 1: 34 (58.6%)	0.919	Chi-square
Prednisone dose (mg/day)	3.38 ± 3.59	3.56 ± 3.86	3.06 ± 3.07	0.569	Wilcoxon
Never used MTX	0: 138 (86.8%); 1: 21 (13.2%)	0: 86 (85.1%); 1: 15 (14.9%)	0: 52 (89.7%); 1: 6 (10.3%)	0.572	Chi-square
Cumulative dose of MTX (mg)	2748.37 ± 3582.17	2536.01 ± 2991.22	3099.58 ± 4399.54	0.829	Wilcoxon

Abbreviations: RF—rheumatoid factor, Anti-CCP—Anti-cyclic citrullinated peptide antibody, LSM—liver stiffness measure, CAP—controlled attenuation parameter, BMI—body mass index, ALT—alanine aminotransferase, AST—aspartate aminotransferase, CRP—C-reactive protein, DAS28-CRP—disease activity score, MTX—methotrexate.

**Table 2:** Multivariable logistic regression of NAFLD with HLA-*DRB1* alleles and covariates. Each row is a separate model (HLA-DRB1*01, *04, *03, *07) adjusted for BMI, age, and sex. The first three columns report covariate effects within each NAFLD model, given as log-odds estimates, SE, *z*, and *p*. The right-most column (NAFLD) reports the allele effect as OR (95% CI), together with the corresponding log-odds estimate, SE, *z*, *p*, and the Benjamini–Hochberg-adjusted *p*.

	BMI and NAFLD	Age	Sex (Female vs. Male)	NAFLD
***HLA*** DRB1*01	estimate = 0.23, SE = 0.05, *z* = 4.92, ***p* < 0.001**	estimate = 0.03, SE = 0.02, *z* = 1.24, *p* = 0.213	estimate = −0.03, SE = 0.47, *z* = −0.12, *p* = 0.902	OR = 0.91, (95% CI: 0.62–1.34), estimate = −0.09, SE = 0.19, *z* = −0.46, *p* = 0.644, adjusted *p* = 0.859
***HLA*** DRB1*04	estimate = 0.25, SE = 0.05, *z* = 5.03, ***p* < 0.001**	estimate = 0.03, SE = 0.02, *z* = 1.36, *p* = 0.175	estimate = −0.05, SE = 0.26, *z* = −0.19, *p* = 0.850	OR = 0.70 (95% CI: 0.46–1.05), estimate = −0.35, SE = 0.20, *z* = −1.72, *p* = 0.086, adjusted *p* = 0.343
***HLA*** DRB1*03	estimate = 0.23, SE = 0.05, *z* = 4.85, ***p* < 0.001**	estimate = 0.03, SE = 0.02, *z* = 1.18, *p* = 0.237	estimate = −0.05, SE = 0.25, *z* = 0.21, *p* = 0.837	OR = 1.29, 95% CI: 0.88–2.28), estimate = 0.25, SE = 0.27, *z* = 0.93, *p* = 0.350, adjusted *p* = 0.700
***HLA*** DRB1*07	estimate = 0.23, SE = 0.05, *z* = 4.92, ***p* < 0.001**	estimate = 0.03, SE = 0.02, *z* = 1.27, *p* = 0.206	estimate = −0.02, SE = 0.25, *z* = −0.10, *p* = 0.921	OR = 1.01, 95% CI: 0.53 to 2.04, estimate = −0.01, SE = 0.34, *z* = −0.02, *p* = 0.980, adjusted *p* = 0.980

Abbreviations: NAFLD—nonalcoholic fatty liver disease; BMI—body mass index; OR—odds ratio; CI—confidence interval; SE—standard error; *z*—*z* value; *p*—*p* value; HLA—human leukocyte antigen.

**Table 3:** Associations of PNPLA3, SLCO1B1, and MTHFR variants with NAFLD (binary), CAP (continuous), and fibrosis (binary). Panel A: NAFLD (CAP ≥ 275 dB/m); logistic models. Panel B: CAP as a continuous outcome (dB/m); linear models. Panel C: Fibrosis (LSM > 8 kPa); logistic models. ‘Minimal’ models adjust for age, sex, and BMI. ‘Fully adjusted’ models additionally include waist circumference (WC), triglycerides (TG), total cholesterol (TC), glucose, cumulative methotrexate (MTX) dose, and prednisone use, as applicable. Odds ratios (ORs) and b coefficients are shown with 95% CIs; estimate/SE/*z* or t are given where available. Adjusted *p*-values use Benjamini–Hochberg control. NR: not reported; NS: not significant (values not reported).

**Panel A—NAFLD (CAP ≥ 275 dB/m): logistic regression**												
**Genotype (Contrast; ref)**		**Model (adjusters)**		**OR** **(95% CI)**		**Estimate**	**SE**	***z***		***p***		**adj. *p***
*PNPLA3*—GC vs. GG (ref = GG)		Minimally adjusted (age, gender, BMI)		1.36 (0.73–2.54)		0.31	0.32	0.98		0.329		0.493
*PNPLA3*—GC vs. GG (ref = GG)		Fully adjusted (age, gender, BMI, waist circumference, triglycerides, total cholesterol, glucose, cumulative methotrexate dose, prednisone use)		1.34 (0.62–2.88)		0.29	0.39	0.76		0.451		0.699
*PNPLA3*—CC vs. GG (ref = GG)		Minimally adjusted		1.04 (0.54–1.99)		0.04	0.33	0.13		0.896		0.949
*PNPLA3*—CC vs. GG (ref = GG)		Fully adjusted		1.16 (0.54–2.46)		0.15	0.38	0.39		0.699		0.699
***Covariate***												
BMI (per kg/m^2^)		Minimally adjusted		1.27 (1.16–1.40)		0.24	0.05	4.90		<0.001		—
Waist circumference (per cm)		Fully adjusted		1.06 (1.01–1.12)		0.06	0.03	2.31		0.021		—
**Panel B** **—** **CAP (continuous, dB/m): linear regression**												
**Genotype (contrast; ref)**	**Model (adjusters)**		**β (95% CI), dB/m**			**SE**	**t**		***p***		**adj. *p***	
*SLCO1B1*—AG vs. AA (ref = AA)	Minimally adjusted (age, gender, BMI)		3.44 (−16.8 to 23.7)			10.2	0.34		0.738		0.745	
*SLCO1B1*—GG vs. AA (ref = AA)	Minimally adjusted		3.40 (−17.2 to 24.0)			10.4	0.33		0.745		0.745	
*MTHFR*—CT vs. CC (ref = CC)	Minimally adjusted		14.7 (2.6 to 26.9)			6.15	2.40		0.018		0.071	
*MTHFR*—TT vs. CC (ref = CC)	Minimally adjusted		3.96 (−15.7 to 7.82)			5.96	−0.67		0.507		0.745	
*MTHFR*—CT vs. CC (ref = CC)	Fully adjusted (+ cumulative MTX dose only)		15.8 (1.7 to 29.9)			7.12	2.22		0.028		0.319	
*MTHFR*—CT vs. CC (ref = CC)	Fully adjusted (cumulative MTX dose, waist circumference, triglycerides, total cholesterol, glucose, prednisone use)		14.6 (0.69 to 28.6)			7.04	2.08		0.039		0.319	
*MTHFR*—TT vs. CC (ref = CC)	Fully adjusted (full set as above)		−4.84 (−18.4 to 8.69)			6.83	−0.71		0.48		0.994	
*SLCO1B1*—AG vs. AA (ref = AA)	Fully adjusted (full set)		−3.99 (−24.8 to 16.8)			10.5	−0.38		0.704		0.994	
*SLCO1B1*—GG vs. AA (ref = AA)	Fully adjusted (full set)		−6.62 (−28.2 to 15.0)			10.9	−0.61		0.545		0.994	
**Panel C—Fibrosis (LSM > 8 kPa): logistic regression**												
**Genotype (contrast; ref)**	**Model (adjusters)**		**OR** **(95% CI)**		**SE**		***z***		***p***		**adj. *p***	
*SLCO1B1*—AG vs. AA (ref = AA)	Minimally adjusted (age, gender, BMI)		1.04 (0.38 to 4.12)		0.564		0.08		0.939		0.992	
*SLCO1B1*—GG vs. AA (ref = AA)	Minimally adjusted		0.87 (0.29 to 3.38)		0.578		−0.24		0.807		0.992	
*MTHFR*—CT vs. CC (ref = CC)	Minimally adjusted		363 (2.2 × 10^−9^ to 6.1 × 10^65^)		509		0.01		0.991		0.992	
*MTHFR*—TT vs. CC (ref = CC)	Minimally adjusted		164 (4.8 × 10^−12^ to NA)		509		0.01		0.992		0.992	

Abbreviations: CAP—controlled attenuation parameter; LSM—liver stiffness measurement; BMI—body mass index; MTX—methotrexate; OR—odds ratio; CI—confidence interval; SE—standard error; *p*—*p* value; *z*—*z* value; NAFLD—Nonalcocholoic fatty liver disease; *SLCO1B1*—solute carrier organic anion transporter family member 1B1; *MTHFR*—Methylenetetrahydrofolate reductase; *PNPLA3*—patatin-like phospholipase domain-containing 3; genotypes: homozygous CC, GG, AA, TT, heterozygous GC, AG, CT.

## Data Availability

The data used in this study is available from the corresponding author upon reasonable request. Due to the sensitive nature of the data, additional approval by our institution may be required.

## Abbreviations

The following abbreviations are used in this manuscript:

NAFLD	Nonalcoholic fatty liver disease
RA	Rheumatoid arthritis
MTX	Methotrexate
BMI	Body mass index
CHOL	Cholesterol
TRIG	Triglycerides
RF	Rheumatoid factor
ACPA	Anti-citrullinated protein antibody
AST	Aspartat aminotrasferase
ALT	Alanine aminotransferase
WC	Waist circumference
HC	Hip circumference
*MTHFR*	Methylenetetrahydrofolate reductase
*SLCO1B1*	Soluble transporter of organic anions 1B1
*PNPLA3*	Patatin 3-like phospholipase domain-containing protein
MHC	Major histocompatibility complex
*HLA*	Human leukocyte antigen
CAP	Controlled attenuation parameter
LSM	Liver stiffness measure
GI	Gastrointestinal
PCR-SSO	Polymerase chain reaction–sequence-specific oligonucleotide
PCR-SSP	Polymerase chain reaction–sequence-specific primer
PCR-RFLP	PCR–restriction fragment length polymorphism
